# Liquid biopsy in pancreatic cancer: the beginning of a new era

**DOI:** 10.18632/oncotarget.24809

**Published:** 2018-06-01

**Authors:** Dipesh Kumar Yadav, Xueli Bai, Rajesh Kumar Yadav, Alina Singh, Guogang Li, Tao Ma, Wei Chen, Tingbo Liang

**Affiliations:** ^1^ Department of Hepatobiliary and Pancreatic Surgery, The Second Affiliated Hospital, Zhejiang University School of Medicine, Hangzhou 310009, China; ^2^ Department of Pharmacology, Gandaki Medical College, Tribhuwan University, Institute of Medicine, Pokhara 33700, Nepal; ^3^ Department of Surgery, Bir Hospital, National Academy of Medical Science, Kanti Path, Kathmandu 44600, Nepal

**Keywords:** liquid biopsy, pancreatic cancer, circulating tumor cells, circulating tumor nucleic acids, exosomes

## Abstract

With dismal survival rate pancreatic cancer remains one of the most aggressive and devastating malignancy. Predominantly, due to the absence of a dependable methodology for early identification and limited therapeutic options for advanced disease. However, it takes over 17 years to develop pancreatic cancer from initiation of mutation to metastatic cancer; therefore, if diagnosed early; it may increase overall survival dramatically, thus, providing a window of opportunity for early detection. Recently, genomic expression analysis defined 4 subtypes of pancreatic cancer based on mutated genes. Hence, we need simple and standard, minimally invasive test that can monitor those altered genes or their associated pathways in time for the success of precision medicine, and liquid biopsy seems to be one answer to all these questions. Again, liquid biopsy has an ability to pair with genomic tests. Additionally, liquid biopsy based development of circulating tumor cells derived xenografts, 3D organoids system, real-time monitoring of genetic mutations by circulating tumor DNA and exosome as the targeted drug delivery vehicle holds lots of potential for the treatment and cure of pancreatic cancer. At present, diagnosis of pancreatic cancer is frantically done on the premise of CA19-9 and radiological features only, which doesn't give a picture of genetic mutations and epigenetic alteration involved. In this manner, the current diagnostic paradigm for pancreatic cancer diagnosis experiences low diagnostic accuracy. This review article discusses the current state of liquid biopsy in pancreatic cancer as diagnostic and therapeutic tools and future perspectives of research in the light of circulating tumor cells, circulating tumor DNA and exosomes.

## INTRODUCTION

Pancreatic cancer (PC) remains one of the most deadly malignancies with an overall five-year survival probability less than 7% in all stages combined [1]. Moreover, it is the fourth leading cause of cancer-related death in the United States, with 53,670 new cases and estimated 43,090 deaths in 2017 [2]. In China, the estimated incidence and death of the PC is 90,100 and 79,400, respectively [3]. Surgical resection is only the main curative treatment; in any case, because of late-presenting clinical features, roughly 30 to 40 percent have the locally advanced disease and another 40 percent have a metastatic tumor at the time of diagnosis. Thus, palliative chemotherapy remains the main treatment option for most of these patients [4–6].

Recently, advances in understanding of the molecular pathology of the PC have given hope to new therapeutic approaches; however, according to recently published systematic review, lack of clinical meaningful trials in the past 25 years might be a reason behind the failure to achieve improvements in early diagnosis, management, and prolongation of overall survival of the PC [7].

With growing research, it is now well understood that the PC is a genetic disease, with complex mutation of cancer genes, and the progression of cancer is characterized by high heterogeneity [8–10]. In earlier studies, KRAS, TP53, CDKN2A, and SMAD4 have been identified as recurrently mutated genes in PC [11, 12]. These discoveries have enhanced our understanding of the molecular pathology of the PC. Recently, genomic analyses have identified different molecular subtypes of PC based on the expression of transcriptional profiles and the structural variations [8, 13–15]. Thus, it's very crucial for early detection of mutant genes to identify PC and its subtypes, for effective management strategy of the disease. In the past decades, numerous studies have shown the potential clinical utility of liquid biopsy, such as circulating tumor cells (CTCs), circulating tumor DNA (ctDNA), and circulating tumor exosomes for various cancers, including PC [16–21]. These promising markers serve as a unique approach for early detection, monitoring and managing disease states. In recent years, noninvasive disease monitoring technology has witnessed an extraordinary explosion of research in the field of liquid biopsy since circulating cell free DNA (cfDNA) was first revealed in body fluids by Mandel and Metais in 1948 [22]. In this review, we have outlined better understanding of different components of liquid biopsy, especially CTCs, ctDNA and exosomes and their potential clinical utility for PC patients. Moreover, we have also drafted numerous of the rational challenges come across using the liquid biopsy techniques.

## CURRENT STATUS IN DIAGNOSIS OF PC

Recently, studies have suggested that pancreatic cancer takes over 17 years to develop, from initiation of mutation in the gene to metastatic cancer, trailed by death roughly after 2.7 years [6, 23, 24]; therefore, if diagnosed early; it may increase overall survival dramatically, and thus, provide a window of opportunity for early detection. Currently, there is no official PC screening program, a confinement of screening for early PC is the absence of sensitive and specific markers [25]. Most commonly used blood-based tumor biomarkers in clinical practice are carbohydrate antigen (CA) 19-9 and carcinoembryonic antigen (CEA). Besides, CA19-9 is the only one currently recommended for clinical use by the NCCN guidelines for PC [26]. According to a recent meta-analysis, CA 19-9 has satisfying pooled specificity while the poor pooled sensitivity for differentiating benign from malignant pancreatic tumors, the pooled sensitivity and specificity were 0.47 (95% CI: 0.35–0.59), and 0.88 (95% CI: 0.86–0.91), respectively [27]. Additionally, it is not tumor specific and is elevated in many hepatobiliary cancers likewise in benign biliary obstruction [28]. In spite of advances in the molecular pathology of the PC, there is no dependable biomarker, the sensitivity and specificity of these currently used tumor biomarkers are definitely not adequate for the early recognition of PC [29, 30].

At present the diagnosis and staging of the PC to a great extent depends on imaging modalities, including ultrasonography (USG), computed tomography (CT), endoscopic retrograde cholangiopancreatography (ERCP), positron emission tomography (PET), magnetic resonance imaging (MRI), magnetic resonance cholangiopancreatography (MRCP), and endoscopic ultrasonography (EUS) [31–37]. In any case, little metastases are hard to detect regardless of the possibility that blends of these modalities are utilized. What's more, these modalities require costly equipment and specialists which are more challenging [35].

Additionally, histological diagnosis often requires invasive tests before surgery. Moreover, because of the difficult anatomical position of the pancreas, a biopsy is often guided by EUS. To the date, endoscopic ultrasonography with fineneedle aspiration (EUSFNA) remains the gold standard in the workup of patients with PC for obtaining the biopsy, with the pooled sensitivity and specificity of 86.8% and 95.8%, respectively [38]. However, EUS-FNA requires sedation and is associated with the number of complications such as tumor seeding along the biopsy tract, pancreatitis, hemorrhage, bowel perforation and aspiration [39–42]. In addition, due to the dense desmoplastic reaction in a PC, the majority of the tumor mass is made up of stromal cells instead of the epithelial cancer cells. Thus, giving rise to false negative results, necessitating frequent repetitive biopsies [1, 41, 43]. Hence, the diagnosis is compellingly done on the premise of CA19-9 and radiological features only. In this manner, the current diagnostic paradigm for the diagnosis of PC experiences low diagnostic accuracy.

Consequently, it is urgent to develop new and improved strategies which can address all the above obstacles and identify primary tumors at an early and resectable stage with greater diagnostic sensitivity *in vitro*, whereas patients with advanced disease must be preoperatively analyzed to dodge surgical impairments and to choose appropriate treatments to enhance the nature of residual life.

## MOLECULAR PATHOLOGY OF PC

Genomic analyses of cancer show that there is a complex mutational landscape and genetic stability of cancer cells are compromised, and PC is no exception to this [8, 24]. Additionally, various genetic modifications occur during the development of the PC, including an increase in duplicate chromosomal number, genetic diversification, amplifications and homozygous deletions, recapitulation of clonal expansion, clonal selection, a small subset of driver mutations and loss of heterozygosity with or without duplicate number reduction [6, 24, 44–51]. Besides, KRAS, p16, SMAD4, CDKN2A, and TP53, are most commonly mutated genes in the majority of the PC patients, particular KRAS mutations occur in almost 92% of the PC cases [8, 11, 12, 52]. In recent years, genomic analyses have an emphasis on the recognition of somatic mutations and other genetic alterations to identify different molecular subtypes of PC. As a breakthrough, some recent studies have defined subtypes of PC based on the expression of transcriptional profiles and the structural variations [8, 13–15].

Collisson *et al*. [13] classified 3 subtypes of PC 1. Classical- Increased expression of adhesion-associated and epithelial genes, e.g. transmembrane protein 45B (TMEM45B), trefoil factor 1 (TFF1) and mucin 13 (MUC13) 2. Quasi-mesenchymal- Increased expression of mesenchyme-associated genes, e.g. Absent in melanoma 2 (AIM2), glycoprotein m6b (GPM6B) and 5’-nucleotidase ecto (NT5E), and 3. Exocrine like- Increased expression of tumor cell-derived digestive enzyme genes, e.g. Islet-derived 1 beta (REG1B), pancreatic lipase-related protein 2 (PNLIPRP2), and cystic fibrosis transmembrane conductance regulator (CFTR).

Likewise, Moffitt *et al*. [14] proposed 4 subtypes of the PC: 1. Normal stroma (high expression of ACTA2, VIM, and DES) 2. Activated stroma (high expression of ITGAM, CCL13, CCL18, SPARC, WNT2, WNT5A, MMP9, and MMP11) 3. Classical (high expression of genes such as BTNL8, FAM3D, and ATAD4), and 4. Basal-like (activation of genes such as VGLL1, UCA1, and S100A2). However, basal-like and classical subtypes were also seen in both the normal stroma and activated stroma subtypes.

In addition, Waddell *et al*. [15] classified 4 subtypes PC based on the structural variation in the mutational landscape 1. Stable (less than 50 structural variations) 2. Locally rearranged (at the minimum of 50 somatic events in the tumor) 3. Scattered (50 to 200 structural variations), and 4. Unstable (more than 200 structural variations).

More recently, genomic expression analysis by Bailey *et al*. also defined 4 subtypes of the PC: 1. Squamous (TP53 and KDM6A) 2. Pancreatic progenitor (FOXA2/3, PDX1, and MNX1) 3. Immunogenic (upregulated immune networks), and 4. Aberrantly differentiated endocrine exocrine (KRAS, NR5A2, RBPJL, NEUROD1, and NKX2-2) [8].

Nonetheless, the interpretation of this molecular subtyping into the clinical setup has been questioned by conflicting outcomes between these studies. Therapeutic agents that can target these subtypes of the PC and their altered genes or their associated pathways may assume a crucial part in the success of precision medicine for the treatment of the PC [53–56].

## LIQUID BIOPSY AS A GAME CHANGER

**Figure 1:**
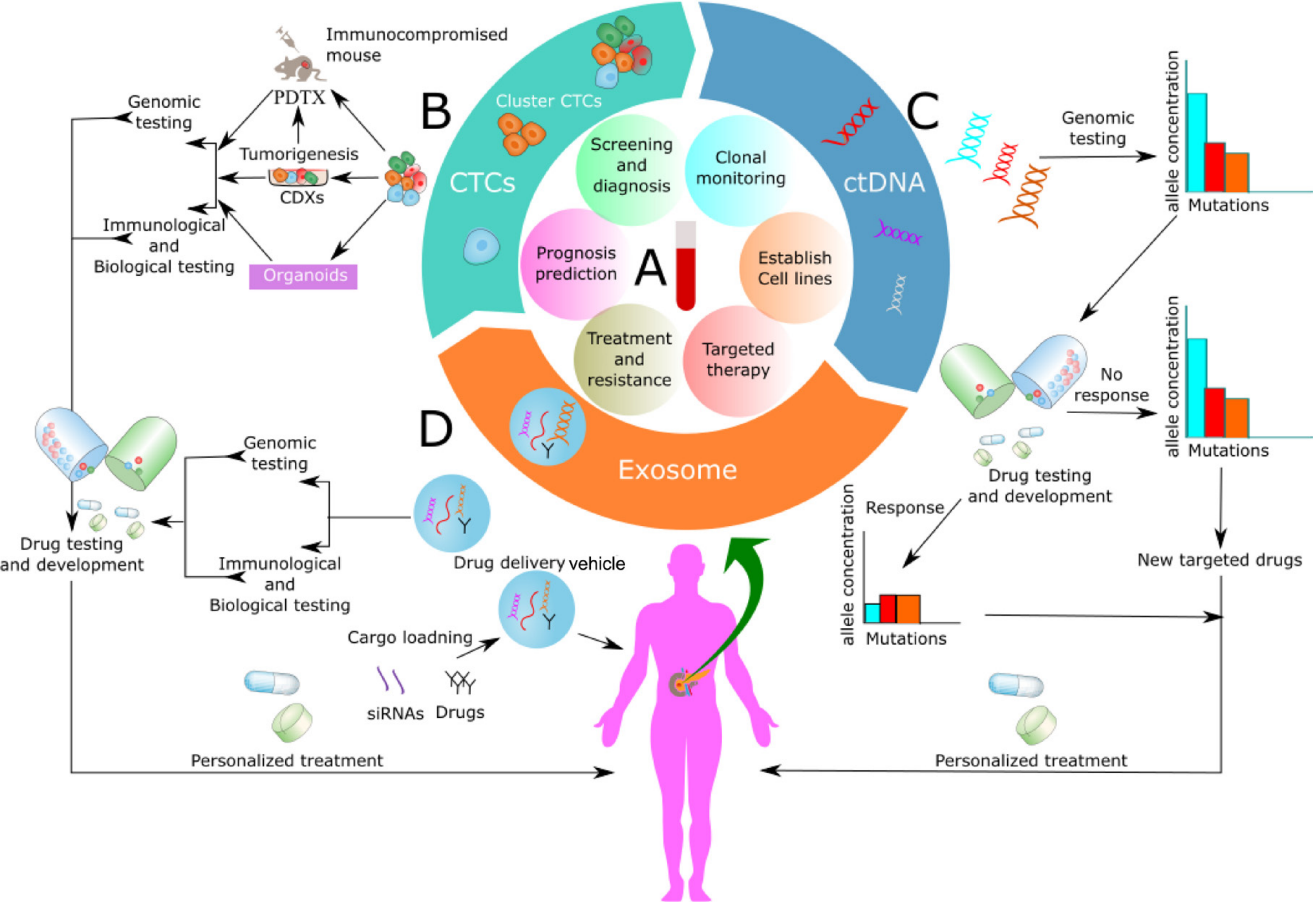
Application of circulating biomarkers (**A**) Application of blood-based liquid biopsy analysis over the span of pancreatic cancer management, peripheral venous blood is collected from the patients for isolation of circulating tumor cells (CTCs), circulating tumor DNA (ctDNA) and exosomes. These circulating biomarkers may be applied to guide initial diagnosis, treatment monitoring or planning, prognosis prediction and developing a new targeted therapy for patients with pancreatic cancer. (**B**) Functional studies with CTCs and development of CTC— derived xeno-grafts (CDXs), patient-derived tumor xeno-graft (PDTX) and 3D organoids model from CTCs for dynamic monitoring of PC and development of new targeted drugs after its molecular characterization and genomic analysis. (**C**) Clinical application of ctDNA as a tool for therapy monitoring. ctDNA can be obtained from plasma for genomic analysis, drug testing and use in personalized medicine according to the genomic and epigenomic alteration. (**D**) Clinical use of exosomes for drug development after genomic and immunological testing. Moreover, use of exosome as a drug delivery vehicle where it can be loaded with drugs, siRNAs, gene etc.

Recently, analysts at The University of Texas MD Anderson Cancer Center have indicated that PC is ready for investigation with a liquid biopsy [57]. A liquid biopsy is simple and painless, minimally invasive sampling and analysis of liquid biomarkers that can be isolated from body fluids, primarily blood [19, 58]. Moreover, liquid biopsies have turned out to be all the more clinically valuable in recent years due to the ability to pair tests on circulating tumor cells (CTCs), circulating tumor nucleic acids (ctNAs) and tumor-derived exosomes with genomic tests [58–61] (Figure 1A). Interestingly, a liquid biopsy can characterize tumor biomarkers, similar to tissue biopsy, which allows early detection of disease, real-time evaluation of metastasis, treatment monitoring, empowers examination of primary tumors, and metastases. Additionally, it enables evaluation of tumor, heterogeneity, cancer dormancy, and monitoring of tumor progression along with prognosis [58, 60, 62–66]. Recently, liquid biopsy that identifies epidermal growth factor receptor (EGFR) gene mutations in non-small cell lung cancers have been approved by FDA [67, 68].

## CIRCULATING TUMOR CELLS (CTCS)

Circulating tumor cells (CTCs), represent tumor cells that contain a heterogeneous population of cells, including apoptotic tumor and viable tumor cells that have cast off into the circulation or *lymphatic* vessels from a primary or metastatic tumor and are transported around the body by undergoing phenotypic changes that are accompanied by a process called as epithelial-mesenchymal transition (EMT) [69–73]. Evidence now suggests that the tumors have ability to make their own blood vessels when they reach the size of 1–2 mm^3^ by inducing angiogenesis or through vasculogenic mimicry, the blood vessels composed of endothelial cells and tumor cells. However, vasculogenic mimicry forms blood vessels without endothelial cells. In fact, they are a mosaic blood vessel which allows substantial shedding of tumor cells into the circulation [74–80]. Likewise, stromal proteins like VEGF and MMP-9 have been known to stimulate angiogenesis in PC [81–83]. Consequently, it has been found that on an average metastatic cancer patient has in the vicinity of 5 and 50 CTCs for approximately every 7.5 ml of blood [84–87]. Nonetheless, the number of CTCs varies between tumor types [88]. CTCs are found in both peripheral blood and bone marrow; additionally, CTCs in the bone marrow are generally called as disseminated tumor cells (DTCs) [89–91]. Further, understanding the CTCs biological properties have demonstrated that the CTCs are involved in the distant organ colonization and metastatic spread of cancer [92–96]. What's more, there has also been growing interest that mobilization of viable tumor cells from the site of primary tumor induced by a therapeutic or diagnostic intervention like surgery, radiation, chemotherapy or tissue biopsy may promote metastasis [97]. To comprehend this process, a team of researcher performed a tumor self-seeding mouse model experiment whereby tumor recurrence intervened by CTCs was examined utilizing human colorectal, melanoma and breast cancer cell lines, and found that tumor-derived cytokines IL-6 and IL-8 act as CTC attractants which were mediated by MMP1, FSCN1, and CXCL1 genes expressed on CTCs to promote infiltration [98]. However, it is not necessarily always associated with metastasis, as only 0.01% of CTCs takes part in metastasis [97, 99, 100]. Moreover, highly sensitive, single cell investigation showed marked heterogeneity of individual CTCs for protein expression and localization, and the CTCs reflected the character of both the primary biopsy and the transformations seen in the metastatic sites [101, 102], which corresponds with the evidence of “seed and soil” hypothesis [103–105].

Amassing proof has demonstrated that CTCs can be utilized as a biomarker to non-invasively supervise cancer progression and provide direction to monitor the treatment [106–108]. However, the American Society of Clinical Oncology (ASCO) fails to recommend the use of CTCs as a tumor marker for breast cancer, in the lack of strong evidence and conflicts of opinion between the experts [109, 110].

Nevertheless, CTCs detection, identification, enumeration, and molecular characterization are very challenging. Since, CTCs are uncommon in peripheral blood of patients (that is, 1–100 CTCs among billions of normal cells) with a half-life between 1 to 2.4 hours and due to its fragile nature, it tends to degrade when collected in standard blood collecting tubes [111–116].

CTCs being a heterogeneous population of cells, CTCs can be positively or negatively enriched on the basis of 1. Physical characteristics and 2. Immunologic or biological characteristics. Physical features include; cell diameter >15 μm, nuclear-cytoplasmic ratio >0.8, electric charges, deformability, the hyperchromatic nuclei, sunken thickened nuclear membrane, and nucleus side-shift/large nucleoli/abnormal nuclear division. Whereas, cell surface protein expression, cell surface markers, RNA, and DNA signatures are used Immunologic or biological characteristics [117–123]. The most common immunologic feature of CTCs exhibits, anti-CD45 antibody (for leukocytes), anti-vimentin antibody (mesenchymal marker), anti-CK8/18/19 antibody and the epithelial cell adhesion molecule (EpCAM) (epithelial marker) and contain a nucleus that binds to the nucleic acid dye 4’, 6-doamidino-2-phenylindole (DAPI) [117, 121]. Further, cytokeratin negative (CK-) CTCs are cancer stem cells (CSCs) that undergoes EMT. Moreover, CK-CTCs have a high potential for metastasis, and are the most resistant type of CTCs and importantly express genes associated with cancer [124, 125]. Similarly, CTCs that are undergoing apoptosis are called apoptotic CTCs, that can be detected by Epic Sciences technology that recognizes nuclear fragmentation or cytoplasmic blebbing associated with apoptosis, and measuring the ratio of viable CTCs and apoptotic CTCs may help in treatment monitoring [124, 126, 127].

Physical properties-based technologies such as ISET (isolation by size of epithelial tumor cells), ScreenCell, ApoStream™, density gradient centrifugation are used to detect, capture, and isolate CTCs. However, size-based selection strategies abuse the fact that CTCs are notably larger in size than normal blood cells [128, 129]. Importantly, nevertheless, this strategy probably results in substantial loss of CTCs which neglects the small CTCs that are cytokeratin positive and CD45 negative, and with similar sizes and shapes to white blood cells. Critically, these small CTCs have cancer-specific biomarkers that distinguish them as CTCs. Additionally, small CTCs have been found in dynamic illness and differentiation into small cell carcinomas, which often require an alternative treatment [130]. CTC cluster containing three or more CTCs is characterized as circulating tumor microemboli (CTM) [121, 129]. Moreover, CTM has high metastatic potential compared to single CTC [131–133]. Lately, researchers involved in an animal experimental model found that a thrombolytic agent like urokinase can prevent the formation of CTM and further prolong overall survival approximately by 20% compared to control. Additionally, they also concluded that CTM mobilizes at a slower rate than the single CTC due to vessel wall adhesion [133].

In the recent years, several platforms have been established for segregation of CTCs that consider both positive and negative enrichment based on physical and immunological features consolidated within the same device [112, 118, 128, 129, 134–147]. A complete outline of these strategies is beyond the scope of this article. Despite these many platforms, CellSearch™ remains only the gold standard and approved by FDA for all the CTCs detection strategies [148–150]. In particular, this CellSearch™ strategy is dependent on the expression of epithelial markers by the CTCs, more specifically the Epithelial Cell Adhesion Molecule (EpCAM) [150, 151]. On the contrary, with respect to recent finding EpCAM based strategy fails to detect CTCs with low EpCAM expression and CK-CTCs, as CTCs tend to lose their epithelial antigens by the EMT process [152–156]. In addition, it has also been recently revealed that EpCAM- negative CTCs are highly aggressive and invasive [154, 157]. Since CellSearch™ method is based on the idea that CTCs do not express the leukocyte antigen CD45, this method also neglects the fact that CTCs can directly attach to platelets and immune cells and thus accounts to be CD45-positive, which further evade immune surveillance that results in clonal expansion and metastatic [158]. In this manner, the CellSearch™ method may underestimate those CTCs that are highly aggressive and invasive [154].

Following detection and isolation of CTCs, the harvested tumor cells are studied for its genetic and biological features. Various molecular techniques, such as immunocytochemistry (ICC), fluorescence *in situ* hybridization (FISH), immunophenotyping, microarray, quantitative reverse transcription-polymerase chain reaction (qRT-PCR), droplet digital PCR (ddPCR), co-amplification at lower denaturation temperature-PCR (COLD-PCR), next-generation sequencing (NGS), beads, emulsion, amplification and magnetic (BEAMing), and whole genome amplification, to mention but a *few* have been commonly performed [159–170]. Despite advancement in these molecular techniques, a genetic study of CTCs still faces challenges of sensitivity and specificity. In particular, digital PCR-based technology is able to screen genetic variations at a very low frequency of 0.01% [171]. However, it only permits monitoring of known mutations and limited numbers of foci [172–175]. Bearing in mind, tumor cells change their mutation under the pressure of therapy or in the midst of tumor sub-clones; therefore, digital PCR-based technology may miss important information during the monitoring process. In addition to this, DNA sequencing of single CTCs for whole genome analysis requires obtaining adequate amounts of DNA and requires institutionalization [176–178]. Moreover, for RNA sequencing strategies larger blood volume is required to obtain an adequate amount of CTCs. What's more, CTCs needs to be captured rapidly in order to avoid RNA degradation. Hence, it's not suitable for cancer screening at the moment [163].

To enhance sensitivity and specificity despite the heterogeneity of CTCs, innovative strategies have to be developed, that can consolidate physical, immunological and genetic analyses together to ease the detection, isolation, enrichment and molecular characterization of CTCs [179].

### Potential clinical utility and research model of CTCs in PC

Currently, the clinical avail of CTCs analysis remains debatable in the PC. To date, numerous analysts have attempted to identify CTCs in patients with PC and have shown its potential clinical utility utilizing different methodologies and with varying results (Supplementary Table 1) [84, 180–190]. Notably, some studies showed that CellSearch™ has a lower CTCs detection rate for PC patients with the sensitivity and specificity of 55.5 % and 100 %, respectively. Additionally, these studies also revealed that CTCs could only be found in malignant pancreatic tumor and CTCs positive patients have a significantly shorter overall survival. However, CTCs detected in these studies failed to correspond with tumor stage [84, 180, 182, 190]. Interestingly, in a study by Zhou *et al*. proposed that the integrated identification of c-Met, h-TERT, CK20, and CEA could be used as an indicator for CTCs in the circulation of a PC patient, which can be detected by combined use of immunomagnetic separation and RT-PCR, and thus, improving the specificity and sensitivity to 100%. Moreover, the positive expression of C-MET, CK20, and CEA was found to be closely correlated with tumor stage [189].

Ankeny *et al*. found that the numbers of CTCs detected from PC was able to differentiate different stages of disease as a useful biomarker and showed 100% similarity for KRAS mutation subtype between primary tumor and CTCs [187]. A meta-analysis comprising 623 patients with different stages of a PC revealed that the patients with positive CTCs had poor progress free survival (PFS) (HR=1.89, 95 %; CI=0.88–2.08, P<0.001) and overall survival (HR=1.23, 95 % CI=1.25–4.00, P<0.001) than those with the CTC-negative patients, suggesting CTCs may be a promising biomarker for the diagnosis and prognosis of a PC [191].

In a study by Yu *et al*., CTCs was isolated by the ^Hb^CTC-Chip microfluidic device from genetically engineered KPC mice and CTCs were subjected to single molecule RNA sequencing, they identified overexpression of the WNT2 gene in CTCs, which prevent anoikis, anchorage-independent sphere formation, and surge metastatic tendency *in vivo*. These findings were supported in CTCs investigated from 5 of 11 patients with PC. Thus, this study proposed that the molecular study of CTCs may recognize patient drug targets [192].

In spite of the fact that evidence indicates the abundance of tumor cells in the blood of patients with PC has prognostic value, and that CTC numbers can be used as a biomarker for diagnosis, staging of a PC before treatment, and can be prescient of response to therapy after treatment and, consequently, treatment results. These results must be considered with vigilance; however, because CTC numbers are highly variable between different CTC detection platforms, and are subject to favoritism relating to the variety of detection methods used. Thus, the quantity of CTCs that can be detected is therefore highly dependent on how the platform characterizes a cell as a CTC. Nevertheless, this impediment is likely to be overcome by consolidating different technologies to enhance analysis performance. Apart from the difference in results between CTCs detection platforms, some other hypothesis has been postulated for reasons behind low CTCs in the PC 1. The blood flow in the PC is notably compromised in contrast to that of the normal pancreas [193]. Thus, less number of CTCs shed into the circulation 2. The moderately low CTCs number reported in PC may be a consequence of CTCs sequestration by the liver as blood advances through the portal circulation into the systemic circulation [190, 194].

In contemporary research, the development of cell lines from CTCs is a motivating novel field. Recently, different groups reported CTCs developed cell lines *in vitro* from patients with breast, mesothelioma, esophageal, bladder, lung, and colon cancer [195–200]. However, some researchers found it to be demanding and reported that CTCderived xenografts (CDXs) foresee therapeutic response conflictingly in many cancers, including the PC [195, 201–204]. Interestingly, this has been confirmed by recent research that pluripotent stem cells cultured in the lab acquire new mutations all the time, especially in TP53 gene [205], and this might be a suggesting reason that why CDXs doesn't correspond to genetic mutations to that of the primary tumor, suggesting that research must be careful while genetic characterization of CDXs. By contrast, direct inoculation of CTCs into immune-compromised mice has met with appreciable success in lung cancer and showed to mirror the response of chemotherapy in CDXs model to that of donor patient. Additionally, it also exhibited comparable genetic profile between isolated CTCs and CDXs model [206]. CDX models have been developed for breast and prostate cancer [207–209]. More interestingly, Gao and colleagues [210] successfully cultured 3D organoids system from CTCs isolated from patients with advanced prostate cancer, which they aim to recapitulate the different subtypes prostate cancer. Likewise, some group of researchers has successfully isolated CTCs from patient-derived tumor xeno-graft (PDTX) models in some cancers, including the PC, and identified its potential clinical utility [211–213]. Perhaps, a standout amongst the most energizing utilizations of CTCs lines is that CDXs and organoids system model may bolster choice of targeted therapies, tracking cancer genetic and epigenetic modifications, and may evolve as an instrumental device for new drug development (Figure 1B). While initial studies using these models are promising, but it needs to be validated with further researches. The primary limitation of these models might be the selection method used for CTC enrichment. In a separate proof-of-concept study, Yu *et al*. created a pharmacogenomic (PGx) model to predict treatment response of a PC patient to chemotherapy regimens based on the genetic mutations in CTCs, and found that PGx profiling of CTCs can forecast treatment response, additionally they found clinical advantage for the patients treated with sensitive chemotherapy regimens versus insensitive chemotherapy regimens in regard to progression free (10.4 mo vs. 3.6 mo; *P* < 0.0001; HR, 0.14) and overall survival (17.2 mo vs. 8.3 mo; *P* < 0.0249; HR, 0.29) [214].

The big question remains: *Can these CTCs derived xeno-graft and organoids models give a mirror image of a PC?*

## CIRCULATING TUMOR NUCLEIC ACIDS (CTNAS)

Circulating tumor nucleic acids (ctNAs) composed of circulating tumor DNA (ctDNA), mRNA and microRNA (miRNA) that are released and circulate in the blood of cancer patients, and changes in the levels of ctNAs in the circulation have been associated with tumor burden, tumor stage, vascularity, cellular turnover, response to therapy, and metastasis [17, 215–217]. At present, the potential clinical utility of cell free RNA (cfRNA) is debatable. A comprehensive discussion of cfRNA is beyond the scope of this article, and this topic has been well documented elsewhere [218]. Moreover, miRNAs are most copious circulating RNA and are also carried in exosomes; thus, this topic has been covered later on with regards to exosomes in this article.

## CIRCULATING TUMOR DNA (CTDNA)

It has been postulated that cell free DNA (cfDNA) can originate directly from the viable tumor cells or from CTCs by apoptosis, necrosis, autophagy, micro-environmental stress, mitotic catastrophe, trauma, and treatment procedure [17, 219–226], others include viruses, such as EBV, HPV and hepatitis B virus [227–229]. Moreover, cfDNA is regarded as ‘circulating tumor DNA’ (ctDNA) after mutations in cfDNA in cancer; hence, information regarding the origination and release of ctDNA may provide insight to clinicians about their possible involvement and nature of the disease. Of these, many studies have shown that ctDNA conveys genomic and epigenomic modifications indistinguishable to those of tumor cells [230]. Studies have demonstrated that cfDNA is cleared from the circulation by means of nuclease activity and renal clearance [231–233]. Additionally, some cfDNA that are taken up by the liver and spleen are degraded by macrophages [234, 235]. Studies in both human and mice have shown that most of the apoptotic cfDNA fragments are measured in the vicinity of 166 and 200 base pairs (bp) with an observed half-life of 16 minutes to 2.5 hours [216, 236–241]. In contrast, necrosis creates higher molecular weight DNA fragments of over 10,000 bp in size due to an inadequate and irregular absorption of genomic DNA [242–245]. However, current isolation strategies are unable to capture long DNA fragments [246]. Indeed, the length of the cfDNA might be clinically valuable, utilized as a surrogate for identification of tumors as ctDNA released from necrosis represents malignant tumor's origin, and increased in the DNA integrity index (ratio of longer fragments to shorter DNA) are seen in most of the malignant tumors [247–249]. However, there are clashing reports in the literature about the origin and composition of ctDNA, a few reports have shown that ctDNAs are shorter than that of apoptotic cfDNAs [238, 250, 251], measuring between 134 and 144 bp [250]. Withal, not all cfDNA originates from cell death; viable cells also release cfDNA as a part of homeostasis [221, 223, 252, 253]. In addition to this, it has also been seen that the activation of lymphocytes can result in the release of large numbers of cfDNA in the absence of apoptosis or necrosis [222, 252, 254]. Moreover, It has been suggested that cfDNA act as a ligand for Toll-like receptor 9 (TLR9) that may inhibit pro-apoptotic caspases by virtue of TLR9-dependent signaling [255]. This signifies a possible immunomodulatory function for cfDNA. These days cfDNA remains to be a hot topic and is widely used for a wide range of research and clinical purposes, including tumor genotyping, early cancer detection, patient prognosis, minimal residual disease monitoring, therapy evaluation, a biomarker in transplant surgery for graft injury, and prediction of allograft rejection [58, 256–268]. In recent years, multiple studies have demonstrated that patients with invasive tumors such as lung, breast, pancreas, colon, hepatocellular, ovarian, prostate, esophageal, and melanoma generally have a high level of ctDNA in their plasma than in healthy individuals [269–274]. Several genomic studies of tumor mutations have analyzed ctDNA to quantify the tumor burden and to detect therapeutic resistance conferring mutations [216, 275–277]. Moreover, a correlation has been set up between the levels of non-mutated cfDNA and mutated cfDNA in circulation and the tumor stage [278, 279]. In addition to this, some studies have also found that mutated cfDNA can lead to therapeutic resistance in cancer several months prior to detection of the tumor by imaging, helping clinicians for therapy evaluation [275, 280, 281].

The study of ctDNA in the plasma basically involves quantification of ctDNA in the circulation using various measurements, for instance mutant allele fraction or mutant allele concentration (that is, copies per milliliter) to estimate disease burden and the detection of genetic aberrations such as somatic mutations, allelic imbalances, genetic polymorphisms, microsatellite alterations, loss of heterozygosity, and methylation [17, 256, 282–288].

There are various methods and technologies used for quantitative and qualitative analysis of ctDNAs, commonly used platforms to name a few are digital PCR (dPCR) [289], droplet digital PCR (ddPCR) [290], BEAMing [291, 292], cancer personalized profiling by deep sequencing (CAPP-Seq) [293], tagged amplicon deep sequencing (TAM-Seq) [276], safe-sequencing (Safe-Seq) [294], duplex sequencing [295], integrated digital error suppression (iDES)-enhanced CAPP-Seq [296], whole-genome sequencing (WGS) [297, 298] and next-generation sequencing (NGS) [299]. Among these NGS holds great expectation for future of genomic analysis.

Regardless of these wide ranges of technologies, extraction, and analysis of ctDNA still face many challenges that need to be addressed before its regular use in a clinical setup. The major challenges are 1. Contamination of ctDNA with a large amount of wild-type cfDNA which are released from lysis of WBC of stored blood in EDTA tubes [300]. Hence, it has been proposed for the utilization of commercially available cell stabilization tubes to prevent or delay the lysis of WBC thereby decreasing the dilution impact of the ctDNA [301]. Additionally, a collection of blood at room temperature and shouldn't freeze more than 2 hours before extracting the plasma for ctDNA analysis, avoid of use heparinised tubes, extraction of ctDNA from plasma rather than serum and a double centrifugation step to remove more cellular debris preceding DNA extraction [300–302]. 2. Low sensitivity and specificity for analysis of ctDNA [303, 304], this could be enhanced by the combination of advanced genomic approaches that have higher sensitivity to identify all ctDNA in the sample, even with small amounts of input material it has been found that multiplexed patient-specific panels in combination with targeted sequencing methods can improve the sensitivity [296, 304, 305]. 3. The expenses of NGS for liquid biopsies are high and the requirement for repeated liquid biopsies for longitudinal study may constrain its use among a substantial group of patients. The quick question emerges: Can patients bear the cost of advanced genomic approaches?

### Potential clinical utility and research model of ctDNA in PC

ctDNA based liquid biopsy brings to the clinic the valuable strength for the success of targeted therapy and precision medicine. Straightaway, understanding of molecular landscapes of PC is vital to guide treatment decisions in clinical practice and with regards to clinical trials. Summary of selected studies using ctDNA for diagnosis, staging, and treatment of pancreatic cancer has been outlined in Table 1 [299, 306–312]. It was Shapiro *et al*. who first reported the presence of ctDNA in PC and exhibited that ctDNA is markedly elevated in PC patients contrasted with healthy controls, and further concluded that ctDNA may serve as a useful diagnostic and prognostic biomarker [269]. Moreover, PC patients with noticeable ctDNA have been appearing to have worse survival and advanced disease stage [313]. In addition to this, ctDNA can be used for real-time monitoring of tumor dynamics, because of its short half-life it can present with the true picture of tumor burden in response to different therapy [216]. Sausen *et al*. demonstrated that the measurement of ctDNA can be used to predict relapse and poor outcome after curative surgery, and recurrence could be detected 6.5 months before radiographic imaging [310]. Moreover, in a recent study, ctDNA was found to be an independent prognostic marker in advanced PC, and the presence of ctDNA was associated with a shorter disease-free survival (4.6 vs.17.6 months) and overall survival (19.3 vs. 32.2 months) after surgery in patients with resectable PC [299]. In a separate study, Tjensvoll *et al*. noted that during chemotherapy of PC patients, changes in mutant KRAS gene level in the circulation corresponded with radiological imaging data and CA19-9 level; moreover, they proposed the utility of ctDNA for monitoring treatment efficacy and tumor progression [312]. These studies demonstrate the potential clinical utility of ctDNA as a prognostic biomarker in PC and further its benefit in monitoring minimal residual disease.

**Table 1:** Studies of circulating tumor DNA (ctDNA) in pancreatic cancer

Study	Pts (N)	Stages	Controls	Time of Analysis	Platform	Markers	Findings	Sensitivity and Specificity
Zill *et al.* 2015 [309]	18	Advanced pancreatobiliary cancers	8 biliary cancer	Post-treatment	NGS	KRAS, TP53, APC, SMAD4, and FBXW7	Mutations were detected in 90.3% of cfDNA. The diagnostic accuracy of cfDNA sequencing was 97.7%, changes in cfDNA coordinated with tumor marker dynamics.	92.3% and 100%
Cheng *et al.* 2017 [308]	188	Metastatic PC	NA	Pre-treatment	NGS and ddPCR	KRAS, BRCA2, EGFR and KDR	The KRAS mutation was detected in 72.3% (136/188) patients. The detection of ctDNA and response to treatment as assessed by CT imaging was 76.9%, the presence of ctDNA provided the earliest measure of treatment in 60% patients.	NA
Berger *et al.* 2016 [307]	24	Metastatic PC	21 IPMN, 38 healthy controls, 26 patients with resected SCAs and 16 borderline IPMN	NA	ddPCR	KRAS	The KRAS mutation was detected in 41.7% (10/24) patients. KRAS mutation was not detected in cfDNA of controls, SCA, and IPMN.	NA
Sausen *et al.* 2015 [310]	77	stage II	NA	Pre-treatment and Post-treatment	NGS ddPCR	NA	ctDNA was detected in the 43% of patients with localized disease at diagnosis, and detection of ctDNA after resection predicts clinical relapse and poor prognosis. Moreover, ctDNA could detect recurrence 6.5 months earlier than with CT imaging.	NA
Henriksen *et al.* 2016 [311]	95	NA	27 without evidence of malignancy, 97 CP and 59 AP	NA	EasyMag platform, PCR	BMP3, RASSF1A, BNC1, MESTv2, TFPI2, APC, SFRP1 and SFRP2	The distinction in mean number of methylated genes in the PC group (8.41 (95% CI 7.62–9.20)) versus the aggregate control group (4.74 (95% CI 4.40-5.08)) was highly significant (*p <* 0.001). Additionally, a diagnostic prediction model (age > 65, BMP3, RASSF1A, BNC1, MESTv2, TFPI2, APC, SFRP1 and SFRP2) had an area under the curve of 0.86 (sensitivity 76%, specificity 83%).	NA
Tjensvoll *et al.* 2016 [312]	14	All stages	29 healthy individuals	Pre-treatment and Post-treatment	PNA-clamp PCR	KRAS	KRAS mutation was detected in 71% of patients with PC. The pre-therapy ctDNA was a predictor of both progression-free and OS. Changes in ctDNA levels corresponded both with radiological follow-up data and CA19-9 levels.	NA
Maire *et al.* 2002 [306]	47	NA	31 CP	Pre-treatment -	PCR and allele-specific amplification	KRAS2	KRAS2 mutation was detected in 22 patients (47%) with PC and in 4 controls with CP (13%) (*P* < 0.002). The combination of KRAS2 and CA19.9 gave a sensitivity and specificity of 98% and 77% respectively.	47% and 87%
Pietrasz *et al.* 2017 [299]	135	All stages	NA	Pre-treatment and Post-treatment	NGS	NA	ctDNA was detected in 48% of patients with advanced PC, and ctDNA emerges as an independent prognostic marker in advanced PC and indicator of shorter DFS and shorter OS when detected after surgery.	NA

PC: Pancreatic cancer; cfDNA: Cell-free DNA; PCR: Polymerase chain reaction; ddPCR: Droplet digital PCR; NGS: Next-generation sequencing; PNA-clamp PCR: Peptide nucleic acid clamping PCR; IPMN: Intraductal papillary mucinous neoplasm; SCAs: Serous cystadenomas; DFS: Disease-free survival OS: Overall survival; CP: Chronic pancreatitis; AP: Acute pancreatitis; CT: Computed tomography; ctDNA: Circulating tumor DNA; NA: Not available.

Since, it is known that over 90% of mutation in PC contains mutated KRAS gene and is considered to be an early event during carcinogenesis [8, 52, 314–316]. Moreover, the mutation occurs most commonly in codon G12D, G12V, and G12R [314, 317, 318]. G12V mutation is significantly associated with shorter survival contrasted to G12D, G12R, and wild-type [319]. Thus, mutated KRAS gene has been a center of surveillance for definitive diagnosis of PC. Discouragingly, chronic pancreatitis also shows the KRAS mutation in 10 to 15% of the cases in cfDNA, and to increase the sensitivity and specificity for diagnosis of PC it has additionally been proposed that combining KRAS mutation and serum CA19-9 level can enhance a sensitivity and specificity by 98% and 77%, respectively [306]. Besides, later on, some studies have shown that methylation analysis of DNA can differentiate PC from chronic pancreatitis and could be used as a potential diagnostic marker for PC [311, 320, 321]. Additionally, it has been found that ctDNA methylation analysis can also detect epigenetic alterations in different cancers, including PC that involves in tumor progression and metastasis [322–325]. Besides, these epigenetic alterations are strongly associated with patient survival [326]. Interestingly, recently it has been proposed that epigenetic alterations in a gene can be reprogrammed genetically or with a pharmacological inhibitor to reverse the epigenetic variations and inhibit their tumor-forming capacity; thus, helping in a finding of attractive therapeutic targets [327]. To the point, the essential question arises: Can ctDNA methylation analysis detect all the epigenetic alterations?

In addition to this, methylation of ctDNA has been found to conceal tissue and cell specific information that may be invaluable in cancer patients to find tissue-of-origin [251, 328], such as in the case of cancers of unknown origin. Of note, methylation analysis of ctDNA is found to be useful in determining the primary location of cancer with a specificity and sensitivity of 99.6% and 97.7%, respectively [329].

Apart from KRAS mutation, in recent years with the development of NGS, increasingly pertinent genetic aberrations have been identified, namely oncogenic BRAF V600E mutation that are observed 3% of PC patients, and that do not acquire a KRAS mutation; similarly, amplification of the MYC oncogene which is remarkably associated with poor prognosis [318]. Moreover, detection of mutations such as RBM10, MLL, MLL2, MLL3, and ARID1A is associated with longer survival [310, 318]. These studies furnish genetic indicators of prognosis and outcome in PC and have suggestions for a new period of therapeutic development (Figure 1C).

In a separate study carried by Zill *et al*. has demonstrated that tumor sequencing was failed in 35% cases of tissue biopsy due to inadequate a tissue sample, in addition to this all mutations were detected in ctDNA similar to that of tissue biopsy. Moreover, they proposed that ctDNA could correlate well with tumor marker dynamics in longitudinal monitoring with a diagnostic accuracy of 97.7%, and with sensitivity and specificity of 92.3% and 100%, respectively [309].

**Figure 2:**
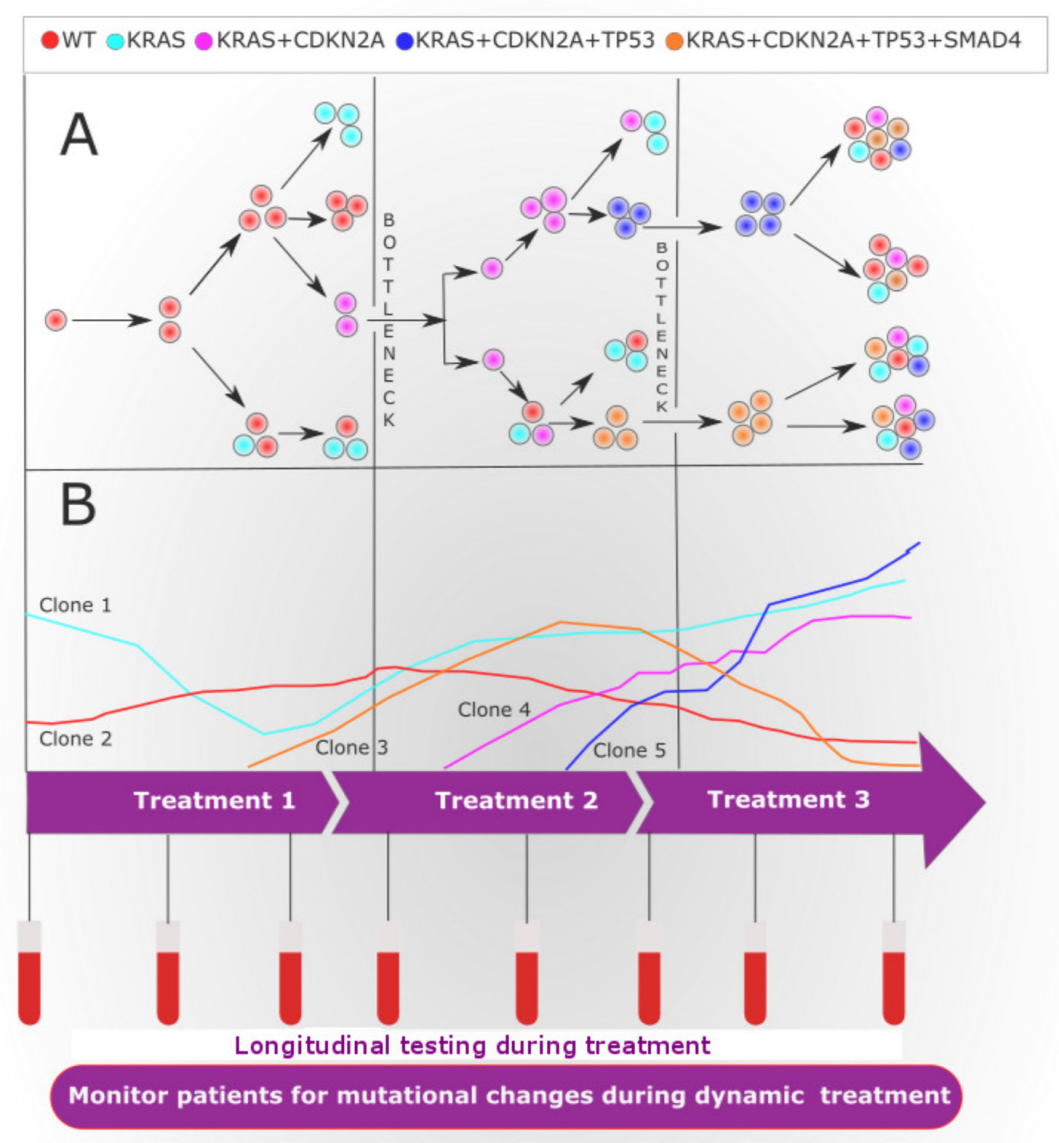
Tumor heterogeneity and clonal evolution during treatment (**A**) Diagram showing the evolutional clonal architecture in pancreatic cancer (PC) at diagnosis and relapse. Of note, at diagnosis, the clonal and subclonal diversity evolved from a common ancestral tumor stem cell. The clonal evolution may follow linear or branched evolution, however, branched evolution is probably more likely to contribute to tumor heterogeneity. Additionally, drug treatment instigates a bottleneck effect, where resistant subclones will survive and proliferate to form a heterogeneous tumor. (**B**) During systemic successive targeted therapy assessed by longitudinal liquid biopsies may identify an actionable genetic alteration, therapy response or progression. In the event that progression is identified, the clinician may be able to switch treatment to target arising clones that carry additional mutations that were identified by the ctDNA analysis. At the start of targeted therapy, all cells in the patient's with PC have actionable genetic mutations (clone 1). The administration of treatment 1 targets the clone 1. longitudinal liquid biopsy analysis demonstrates an initial decrease in the clone 1 during treatment 1, yet uncovers the evolution of new clone (clone 2 and clone 3) causing resistance to treatment 1. The clone 2 and clone 3 can be targeted with treatment 2, where longitudinal liquid biopsy analysis uncovers a decrease in the frequency of resistance clone 2 and clone 3, during this time, however, other genetic alterations clone 4 and clone 5 increases in frequency. These clones 4 and 5 are resistant to treatment 2, yet is sensitive to treatment 3. During treatment 3, the frequency of the clone 4 and clone 5 decreases, while residual earlier resistant clones may persist to give rise to therapeutic resistance.

Intra- and inter-tumor heterogeneity contribute to the development of drug-resistant tumors and failure of treatment [330]. A small genetic clone carrying a drug-resistant mutation within the tumor can extend after the pressure treatment. These genetic clones can be missed by tissue biopsies due to low prevalence or the spatial partition of cells within the tumor [331]. Interestingly, ctDNA can be exploited to monitor dynamic clonal and subclonal evolution in response to the pressure of therapy [332] (Figure 2).

## EXOSOMES

Exosomes are very stable, small cup-shaped, lipid bilayer microvesicles of endocytic origin with a size of 50–150 nm in diameter and density of 1.12–1.19 g/ml [60, 333, 334]. These microvesicles are discharged by all cells, including tumor cells, and originally thought to be that they are like cellular garbage bags [335–337]. However, recent research suggests that exosomes are involved in many physiological and pathological functions and processes such as intracellular communication, inflammation, cell proliferation and regeneration following injury, immune response, lactation, neuronal function, immunothrombosis, diabetes, atherosclerosis, development, and progression of liver disease, neurodegenerative diseases and more recently in cancer [338–352]. Evidence demonstrates that exosomes are available in numerous biologic body fluids; exosomes may in this way be viewed as open indicative biomarkers that hold incredible potential for recognition of numerous disease conditions, including cancer [353, 354]. Interestingly, exosomes are enriched with DNA, proteins, lipids, RNAs, and metabolites that are reflective of the cell types of origin [352, 355–357]. Nonetheless, whole RNA sequence can't be bundled inside one exosome, because of its small size, which was contrasted and retrovirus particles of a comparable size that can just pack 10 kb transcriptome, subsequently, single exosome conveys just a predetermined number of transcripts [358]. Still, exosomes are remarkably abundant in plasma and when segregating the vesicle portion, the vast majority of the RNA sequence can be identified [359, 360]. Recent data from various cell type uncovers that exosomes contain 4,563 proteins, 194 lipids, 1639 mRNA and 764 microRNA [361]. Among the main 20 regularly recognized exosomal proteins, a significant number of the proteins, including CD9, ACTB, CD63, CD81, HSPA8, PKM2, ANXA2, HSP90A1, SDCBP, YWHAE, LDHA, MSN, PDCP6IP, ANXA5, FASN, ACTN4, LDHB, ANXA1, HSPA1A, and YWHA are known to be mutated in multiple cancer types [362]. Additionally, it has been found that some exosomes reveal major histocompatibility complex MHC I and MHC II on their surface, suggesting that they are derived from antigen-presenting cells and might have a regulatory immunological part in cancer biology [363, 364]. Considering, exosomes convey genomic and proteomic materials, thus it has been hypothesized that exosomes secreted by tumor cells take part in the tumor growth, invasion, pre-metastatic niches (PMNs) and metastasis through intracellular communication and escape from immunosurveillance [338, 365–373]. Biogenesis and secretion of exosome within the cell is a complex process which requires different factors like molecular motors (cytoskeleton- kinesins and myosins, polymerisation- actin, dynamin, and microtubules), molecular switches (GTPases, annexins, and flotillin) and the fusion proteins (SNARE proteins and tethering factors), cargo sorting proteins complex (ALIX and TSG101) and finally exocytotic released is promoted by cellular stress or hypoxia in cancer cells [333, 374–377]. Studies from Thery and colleagues reveal that Rab27a and Rab27b act as key downregulators of the exosome secretion pathway, which inhibit secretion of exosomes [378]. These Rab family proteins are thought to be involved in cancer progression and tumor advancement, which provided clues that exosomes have something to do with tumor biology [379]. Moreover, p53 protein, and a p53-regulated gene, TSAP6, have shown to increase production of exosome [380, 381]. Emerging evidence suggested that breast and pancreatic tumor-derived exosomes express integrins (ITGs) on their surface which direct organ-specific colonization by fusing with targeted stromal and cancer cells, thereby forming PMNs within the cancer microenvironment to transmit signals and their cargo, that includes genetic material (that is, DNA, mRNA, and miRNA), metabolites and proteins, by that determining organotropic metastasis [338, 366–368, 373, 382–384]. Subsequently, PMNs requires S100 family proteins for homing of tumor-derived exosomes in targeted organs [373]. Furthermore, tumor-derived exosomal miRNA and protein have a tendency to reprogram and instruct target cells that it fuse with towards pro-inflammatory and pro-metastatic phenotype leading to metastasis [382, 385].

Till the date, numerous of technologies and methods have been used for extraction of exosomes from body fluids which have been well documented elsewhere [386]. Commonly used methods are ultracentrifugation-based isolation, precipitation-based isolation, size-based isolation, immunoaffinity-based isolation, and microfluidics-based isolation [386]. The segregation of tumor-derived exosomes from patients; however, remains challenging due to some of the reasons: 1. Lack of a standardized method for segregation and the absence of specific markers that can differentiate tumor-derived and non-tumor derived exosomes [387]. 2. Failure in isolating large concentration of exosomes, due to contamination from other extracellular vesicles and cellular debris [386]. 3. Time-consuming technology that is hard to implement in routine clinical setup [386].

To overcome these challenges, an institutionalized technique for exosome isolation should be developed sooner rather than later, thus amplifying the significance of research facility based investigations of exosomes in the clinical setting. It is important to reliably approve each of these strategies as per meticulous definitions of exosomes, those laid out by the International Society for Extracellular Vesicles [388].

### Potential clinical utility and research model of exosomes in PC

As the content of exosomes is cell-type specific with an extensive variety of molecular information carried forth from parent cells to secondary cells, exosomes may provide an idiosyncratic ‘signature’ of tumor development and metastatic progression, as well as the metabolic status of the tumor. In spite of the fact, that the mechanism of packaging is yet to be completely comprehended, it has been seen that the metastatic tumor cells show the high ability of packing and cargo secretion (that is, protein, RNA, DNA, and metabolites) in exosome [382, 385]. To date, numerous studies have outlined clinical utility of exosomes as a diagnostic, prognostic and therapeutic tool in PC patients (Table 2) [387, 389–393].

**Table 2:** Studies of circulating tumor exosomes in pancreatic cancer

Study	Specimen type	Platform	Markers	Findings and Conclusion
Que *et al.* 2013 [389]	Serum	Filtration, Ultracentrifugation, and RT-PCR	miR-17-5p, miR-21, miR-155, and miR-196a	There were low expressions of exosomal miR-155 and miR-196a in PC patients. Moreover, there were high expressions of serum exosomal miR-17-5p and miR-21 in PC patients than control groups and high expression of miR-17-5p was significantly correlated with advanced stage of PC.
Kahlert *et al.* 2014 [390]	Serum	Filtration, Ultracentrifugation and WGS	KRAS, p53	Exosomes from PC patients contain >10-kb fragments of double-stranded genomic DNA with detectable mutations in KRAS and p53. In addition, WGS of exosomal DNA can determine genomic DNA mutations for cancer prediction, treatment, and therapy resistance.
Madhavan *et al.* 2015 [391]	Serum	Ultracentrifugation, qRT-PCR, and Flow cytometry	CD44v6, Tspan8, EpCAM, c-Met, CD104, miR-1246, miR-4644, miR-3976, and miR-4306	Serum exosomal miR-1246, miR-4644, miR-3976 and miR-4306 were significantly upregulated in 83% of PC serum-exosomes, but rarely in control groups. Additionally, It was found that a combination of five proteins (CD44v6, Tspan8, EpCAM, MET and CD104) and four miRNAs (miR-1246, miR-4644, miR-3976 and miR-4306) in circulating tumor exosomes could recognize PC from healthy control, chronic pancreatitis and benign pancreatic disease with a sensitivity and specificity of 100% and 80% respectively.
Melo *et al.* 2015 [387]	Serum	Filtration, Ultracentrifugation, qRT-PCR And Mass spectrometry analyses	Glypican-1	Expression of glypican 1 (GPC1) a membrane-bound protein on circulating exosomes of mice and humans with PC can differentiate healthy control and patients with a benign pancreatic disease. Notably, GPC1^+^ exosomes level correlated with tumor burden and the survival of PC patients before and after the surgery with utter sensitivity and specificity. Additionally, circulating GPC1^+^ exosomes of PC patients bear KRAS mutations,
Kanwar *et al.* 2014 [392]	Serum	ExoChip (antigen based)	CD63	Significantly higher exosome capture in PC patients, compared to controls.
Allenson *et al.* 2017 [393]	Whole blood	Ultracentrifugation, Flow cytometry, and ddPCR	KRAS	Exosomal DNA posses KRAS mutations and was detected localized, locally advanced, and metastatic PC patients, respectively. Higher exosomal DNA KRAS mutations were associated with decreased disease-free survival in patients with localized disease.

PC: pancreatic cancer; GPC1: glypican-1; miR: microRNA; ddPCR: Droplet digital polymerase chain reaction; qRT-PCR: Quantitative reverse transcription polymerase chain reaction; WGS: Whole-genome sequencing; EpCAM: Epithelial cellular adhesion molecule; RT-PCR: Reverse transcription polymerase chain reaction.

In a seminal research, Melo *et al*. [387] demonstrated an increased amount of glypican 1 (GPC1) a membrane-bound protein on circulating exosomes of mice and humans with PC can differentiate healthy control and patients with a benign pancreatic disease. Notably, GPC1^+^ exosomes level correlated with tumor burden and the survival of PC patients before and after the surgery with utter sensitivity and specificity. Additionally, circulating GPC1^+^ exosomes of PC patients bear KRAS mutations, and were able to identify pancreatic intraepithelial neoplasia (PanIN) in mice from healthy control even before detectable pancreatic lesion on MRI. Of note, the main limitation of this study was a small sample size. Undoubtedly, these findings should be verified with a larger series of the sample, but the striking evidence provided by Melo and colleagues suggest that GPC1^+^ exosomes may serve as a potential diagnostic and screening biomarker to detect early stages of PC for possible curative surgery. In earlier studies, overexpression of surviving [394], and mislocalization of plectin [395] in exosomes were also proposed as biomarkers for PC. Moreover, it has also recently been found that a higher rate of patients with localized PC showed noticeable KRAS mutations in exosomal DNA than previously revealed for cfDNA, and thus exosomal DNA may act as a complementary DNA source to liquid biopsy [393]. In a research, Madhavan *et al*. outlined that a combination of five proteins (CD44v6, Tspan8, EpCAM, MET and CD104) and four miRNAs (miR-1246, miR-4644, miR-3976 and miR-4306) in circulating tumor exosomes could recognize PC from healthy control, chronic pancreatitis, and benign pancreatic disease with a sensitivity and specificity of 100% and 80%, respectively [391].

Exosomal micro-RNAs (miRNAs) have additionally increased generous consideration in later past years. From the recent studies, the number of exosomal miRNAs including miR-21, miR-17-5p, miR-155, miR-34, miR-196a, miR-181a, miR-181b, miR-138-5p, miR-494, miR-542-3p, miR-31, and miR-205 has been identified and upregulation of these miRNAs has been shown to increase cellular proliferation, angiogenesis promotion, disease progression, metastasis, and chemo-resistance in PC patients [389, 396–403]. Moreover, these studies highlight the potential use of exosomal miRNAs as a diagnostic and prognostic biomarker. Likewise, targeting the exosomal miRNAs might be a potential therapy for PC.

Additionally, it has been found that miRNAs in circulating exosomes are representative of those increased in the primary tumor cells [21]. In a separate study, Ohuchida *et al*. distinguished 24 miRNAs with altered expression in gemcitabine-resistant cells, and furthermore found that patients with high miR-142-5p and miR-204 expression had significantly longer survival times than those with low miR-142-5p and miR-204 expression in the gemcitabine-treated group [404]. Despite the fact that the miRNA levels were determined in paraffin-embedded tissue, this highlights the potential use of tumor-derived exosomal miRNAs as predictors of response to chemotherapy and future use of miRNAs for targeted immune therapy in PC. Moreover, it had been proposed that exosomes miRNAs are derived from living cells, while circulating free miRNAs usually originates from apoptotic or necrotic cells [398], and thus, exosomes miRNAs might have advantages over circulating free miRNAs for monitoring therapy or late stage of PC.

Lyden and colleagues [373] in their recent paper proposed that tumor-derived exosomes integrins (ITGs) can determine organotropic metastasis as discussed in the earlier section of this paper. The consequent analysis demonstrated that liver-tropic pancreatic exosomes expressing ITGαvβ5 could communicate with F4/80+ macrophages and fuse with Kupffer cells in fibronectin rich liver niches. Besides, inhibiting ITGβ5 expression through short hairpin RNAs or hindering their binding by HYD-1/RGD peptides particularly reduced exosome uptake and additionally liver metastasis. This study explains why the liver is the most common site for PC metastasis. Moreover, these outcomes showed that exosomal ITGs may be used as organotropic biomarkers to anticipate organ-specific metastasis in PC patients, and expands our understanding of the organ-specific metastasis mechanisms involvement of exosomes in advancing tumor metastasis. In addition to Lyden results, a study by Costa-Silva *et al*. [385] demonstrated that the PC exosomes can expand liver metastatic burden by transferring macrophage migration inhibitory factor (MIF) to Kupffer cells and by recruiting immune cells to initiate PMNs development in the liver. It is thus proposed that the presence of MIF in exosomes may be a biomarker that can show the likelihood of PC metastasis to the liver and blockage of MIF could prevent liver metastases and may prove to be clinically relevant for the development of new targeted therapies.

The utilization of exosomes as a nucleic acid, gene or drug delivery vehicles (Figure 1D) has increased significant enthusiasm because of their phenomenal biodistribution and biocompatibility [405]. Moreover, the advantage of utilizing exosomes as a drug delivery system lies in the fact that they can be particularly targeted to a specific cell type by engineering exosome-producer cells [406]. Interestingly, past studies have demonstrated that tumor cells secrete more exosomes compared to normal cells [407]. Furthermore, malignant pancreatic tumor cells, with oncogenic RAS have also founded to uptake exosomes more readily through the active induction of macropinocytosis [408, 409], and this could strengthen the use of exosome as an ideal drug delivery vehicle. Recently, Kamerkar *et al*. demonstrated treatment efficacy of engineered exosome (iExosome), where iExosome was able to suppress the PC progression in genetically engineered KTC and KPC mouse models, this study exhibited an approach for direct and specific targeting of KRAS mutation in tumors using engineered exosomes [410]. However, it still needs to be verified in the clinical setting.

## CONCLUSIONS

We have accomplished enormous progress in our understanding of the complex molecular and genetic mechanisms of PC, yet key inquiries stay unanswered for its early diagnosis, staging, treatment monitoring, and management. Taking everything into account, the up and coming era of ‘ liquid biopsy’ will be vital to conclusively build up the clinical relevance of bloodbased genomic profiling. Liquid biopsy methodologies will most likely give enhanced diagnostic and therapeutic outcome. However, a few issues stay to be tackled before application in a clinical setting: 1. Institutionalization of the sample collection methodology in pre-analytical setup, subsequently decreasing pre-analytical errors 2. Institutionalized and strict definition of CTCs, ctDNA, and exosomes for their segregation and analysis is required. 3. Improvement in the sensitivity and specificity of the detection methods by integrating CTCs, ctDNA, and exosomes in one platform. 4. Universal signature from CTCs, ctDNA, and exosomes for differentiating benign from malignant disease and that can cover all phases of cancer along with their subtypes, tumor characteristics, and mutations for the success of precision medicine. 5. Substantial forthcoming clinical trials, including multicenter studies, are expected to approve the clinical essentials for diagnosis, treatment monitoring, and prognosis. The comparison of CTCs, ctDNA, and exosomes is outlined in Table 3.

**Table 3:** Comparison between CTCs, ctDNA and exosomes as liquid biopsy

Comparison	CTCs	ctDNA	Exosomes
**Origin**	Includes apoptotic tumor and viable tumor cells from a primary or metastatic tumor [71, 73].	Includes cfDNA, from the viable tumor cells or from CTCs [17, 219, 221, 224, 225].	Includes DNA, proteins, lipids, RNAs and metabolites and are discharged by all cells including tumor cells [333, 336, 352, 355–357].
**Bio-banked samples used for study**	Frozen plasma, urine and other biofluids cannot be used for study of CTCs [116].	Frozen plasma, urine and other biofluids can be used for study of cfDNA [116, 260].	Frozen plasma, urine and other biofluids can be used for study of exosomes [260].
**Stability**	Unstable [114, 116, 117]	Stable [116]	Very stable [334]
**Genetic materials**	DNA and RNA [117, 122, 123]	DNA [17, 219, 221]	DNA and RNA [355, 357, 359–361]
**Analytic Techniques**	CellSearch [84, 148–150, 180, 182, 190], Microfluidic [187, 181], SE-iFISH [186], MetaCell [184], Immunofluorescence [181, 185, 188], ScreenCell [185], ISET Test [188, 190] etc.	dPCR [289], CAPP-Seq [293], TAM-Seq [276], ddPCR [290, 307, 308, 310], COLD-PCR [167], Safe-Seq [294], NGS [299, 308–310], BEAMing [291, 292], WGA [297, 298] etc.	Ultracentrifugation [387, 386, 389–391, 393], ExoChip [392], Precipitation [386], Size-based isolation [386], Immunoaffinity-based isolation [386], Microfluidics-based isolation etc [386].
**Morphological study and functional study of tumor cells *ex vivo***	Yes [128, 129, 188, 190, 206, 210, 213, 214]	No	No
**Analysis of protein location on tumor cells**	Yes [101, 102]	No	No
**Identification of mutations**	Yes [165, 185, 187]	Yes [306, 319]	Yes [387, 390, 393]
**Identification of epigenetic changes**	Yes [414, 415]	Yes [322–325]	Yes [357]
**Analysis of RNA transcription profiles**	Yes [157, 192]	No	Yes [359–361, 367]
**Proteomics Analysis**	Yes [157]	No	Yes [367, 387, 392]
**Analysis tumor heterogeneity**	Yes [206]	Yes [332]	No
**Use as drug delivery vehicle**	No	No	Yes [406, 410]

DNA: Deoxyribonucleic acid; RNA: Ribonucleic acid; CTCs: Circulating tumor cells; ctDNA: Circulating tumor DNA; cfDNA: Cell free DNA; ISET: Size of Epithelial Tumor; SE-iFISH: Immunostaining-fluorescence *in situ* hybridization; ddPCR: Droplet digital PCR; COLD-PCR: Co-amplifcation at lower denaturation temperature-PCR; NGS: Next-generation sequencing; BEAMing: Beads, Emulsion, Amplifcation and Magnetic; WGA: Whole genome amplifications, messenger RNAs (mRNAs); dPCR: digital PCR; CAPP-Seq: Cancer personalized profiling by deep sequencin; TAM-Seq: Tagged amplicon deep sequencing; Safe-Seq: Safe-sequencing.

In spite of the presence of various challenges, liquid biopsy seems to be ideal diagnostic and therapeutic strategies for PC. So far, in June 2016, a liquid biopsy was approved by the FDA for use in the USA to detect EGFR mutations in plasma ctDNA and entered clinical practice for the management of non-small cell lung cancer (NSCLC) [68]. After its approval, it represents key milestones towards the application of liquid biopsies in personalized clinical oncology.

CTCs seem to have enormous potential for PC, and can be exploited to understand the development of the distant organ colonization and metastatic spread of cancer. Moreover, CTCs can be used to understand the phenotypic changes, plasticity of tumor biology and mutational landscape of tumor by development of PDTX [213], PGx [214], CDXs [206], and 3D organoids [210] models and guide treatment decisions for complex disease like PC [184, 190, 411–413]. However, methylation analysis of CTCs remains largely unexplored, except few studies which have been reported in recent years [414, 415]. Methylation study of CTCs holds a promising future with an exciting result; this may give a new direction to upcoming research.

In the ctDNA arena, ctDNA has offered more an inclusive understanding of a patient's disease. For instance, the total ctDNA concentration can be used for real-time monitoring of tumor dynamics and predicts relapse, poor outcome and shorter disease-free survival after curative surgery [299, 310, 313]. Methylation analysis of ctDNA can detect epigenetic alterations that involve in tumor progression and metastasis [322, 325]. Moreover, it can also differentiate PC from chronic pancreatitis and could be used as a potential diagnostic marker for PC [320, 321]. However, the sensitivity and specificity of ctDNA analysis are struggling and it can be increased by adopting a multi-marker strategy along with integrating it with other biomarkers.

Exosomes provide an enormous understanding about organ-specific colonization and PMNs [373, 385]. Moreover, exosomes can serve as a potential biomarker as its contents are largely derived from the tumors, which are enriched with DNA, proteins, lipids, RNAs, and metabolites. In addition to this, exosomes as drug delivery vehicles offer an important perspective because of its cell-specific nature, excellent biodistribution and biocompatibility [405]. Before these drug delivery systems become a therapeutic reality, it needs to be validated with further researches and large clinical trials.

In general, a liquid biopsy can possibly be used to diagnose PC at an early stage, predict prognosis, monitor PC stage, therapeutic efficacy or resistance, and provide optimal, personalized treatment strategies for patients with PC. This review has endeavored to organize the present advances in liquid biopsy for PC into a solitary idea to establish an effective management plan and implementation of these understandings to bolster energizing zones of research. But the fundamental question remains: Can liquid biopsy become a screening reality for pancreatic cancer?

## SUPPLEMENTARY MATERIALS TABLES




