# Supplementary material for: Liquid biopsy in pancreatic cancer: the beginning of a new era
Source: Oncotarget. 2018 Jun 1;9(42):26900–33. doi: 10.18632/oncotarget.24809 (PMC6003564; doi:10.18632/oncotarget.24809)
Supplement: Supplementary file 1 [file oncotarget-09-26900-s001.pdf]

Liquid biopsy in pancreatic cancer: the beginning of a new era

SUPPLEMENTARY MATERIALS

Supplementary Table 1: Studies of Circulating Tumor Cells (CTCs) in Pancreatic cancer. See Supplementary_Table_1
