# Supplementary material for: Liquid biopsy in pancreatic cancer: the beginning of a new era
Source: Oncotarget. 2018 Jun 1;9(42):26900–33. doi: 10.18632/oncotarget.24809 (PMC6003564; doi:10.18632/oncotarget.24809)
Supplement: Supplementary file 2 [file oncotarget-09-26900-s002.docx]

**Table 1- Studies of Circulating Tumor Cells (CTCs) in Pancreatic cancer**

| **Study** | **Pts (N)** | **Stages** | **Time of Analysis** | **CTC Platform** | **Markers** | **Findings** | **Sensitivity and Specificity** |
| --- | --- | --- | --- | --- | --- | --- | --- |
| Allard et al. 2004 [84] | 16 | NA | Preoperative | CellSearch Immunomagnetically | Morphology, DAPI, CK, and CD4 | The CellSearch platform might be utilized to decide the clinical utility of CTCs. CTCs are extremely rare in healthy subjects and patients with nonmalignant diseases, but present in various metastatic carcinomas with a wide range of frequencies. | NA |
| Zhou et al. 2011 [189] | 40 | All stages | Preoperative | Immunomagnetic  separation | C-MET,  h-TERT, CK20, and  CEA | The sensitivity for detection of CTCs using C-MET, h-TERT, CK20, and CEA was 80 (20/25), 100 (25/25), 84 (21/25) and 80% (20/25), respectively. CK-20 CTCs were found in 1/25 (6.77%) patients with benign disease. | 100% and 93.3% |
| Khoja et al. 2012 [190] | 54 | Stage III or  IV | Preoperative | Size-based; ISET, CellSearch | EpCAM, CK  vimentin, and  E-cadherin | CellSearch detected ≥1 CTC in 40% of patients versus 93% for ISET. ISET found CTCs in higher numbers. ISET isolated additional mesenchymal-type CTCs not captured by CellSearch. | NA |
| Iwanicki et al. 2013 [180] | 40 | NA | Preoperative | CellSearch | CK,CD45,DAPI, and EpCAM | CTCs were detected in 15/27 patients of PC and CTC status did not correlate with metastatic status, lymph node involvement, vascular invasion, size of tumor and CA19-9 serum level. | 55.5 % and 100 % |
| Rhim et al. 2014 [181] | 51 | NA | Preoperative | Microfluidic Immunofluorescence | EpCAM, CK, CD45, DAPI, and PDX-1 | CTCs were found in 8/11 patients of PC, 7/21 patients with cystic lesions and 0/19 patients without cysts or cancer (controls). CTC are present in the circulation of patients before tumors develop may help in risk assessment. CTC status did not correlate with tumor stage and CA19-9 serum level. | NA |
| Earl et al. 2015 [182] | 35 | Resectable, locally advanced and metastatic disease | Preoperative | CellSearch | CK, CD45, DAPI, and EpCAM | CTCs were detected in 7/35 patients of PC, CTC positive patients had a significantly shorter overall survival. CTC detection is promising markers for the management of patients with PC and is usually found in patients with a metastatic disease. | NA |
| Zhang et al. 2015 [183] | 22 | 2 stage I, 10 stage II, 4 stage III and 6  stage IV | Preoperative and Postoperative | Immunestaining and FISH with CEP8 | CK, CD45, DAPI, and CEP8 | CTCs found in 15/22 patients of PC. CTCs count did not signiﬁcantly correlate with disease stage, presence of lymph node metastases, and presence of distal metastases or CA19-9. CTCs positive patients showed metastasis and worse survival rate. CTCs count decreased in 3 days after surgery, but increased in 10 days after surgery in most patients. | 68.18 and 94.87% |
| Bobek et al. 2014 [184] | 24 | 1 stage I, 12 stage II, 8 stage III and 3 stage IV | Preoperative | Size-based separation MetaCell | Cell nuclei and cytomorphology, CK7, CEA, and  vimentin | CTCs found in 16/24 patients of PC. CTCs positivity does not reflect the disease stage. | NA |
| Kulemann et al. 2016 [185] | 21 | NA | Preoperative | ScreenCell, Immunofluorescence | ZEB1 and an epithelial antigen CK | CTCs found in 18/21 patients of PC. Molecular and genetic characterization, including mutations such as KRAS, may prove useful for prognosis. ZEB1 was found almost exclusively in CTC of patients with established metastases. | NA |
| Gao et al. 2016 [186] | 25 | 5 stage I, 8 stage II, 6 stage III and 6 stage I | Preoperative | SE-iFISH platform | CK, CD45, DAPI, and CEP8 | A total of 103 CTCs were detected among the 25 PC patients. CTCs could be detected in PC patients in various stages, whether localized, locally advanced and metastatic. Besides, CTCs have shown the potential implication in predicting prognosis of PC. | 88 % and 90 % |
| Ankeny et al. 2016 [187] | 72 | NA | Preoperative | Microfluidic NanoVelcro CTC chip | CK, CD45, and DAPI | CTCs found in 54/72 patients of PC. A cut-off of ≥3 CTCs in 4 ml venous blood was able to discriminate between local/regional and metastatic disease. CTCs appear to function well as a biomarker for diagnosis and staging in PC. There was 100% concordance for KRAS mutation subtype between primary tumor and CTCs. | 75.0% and 96.4% |
| Poruk et al. 2016 [188] | 60 | NA | Perioperative | ISET, Immunofluorescence | ALDH, CD133, and CD44 | CTCs found in 47/60 patients of pancreatic cancer. ALDH-positive CTCs and triple-positive CTCs were significantly associated with worse survival. ALDH-positive CTCs, triple-positive CTCs, and dual cytokeratin- and CD133-positive CTCs were independent predictors of tumor recurrence. | NA |

**PC:** Pancreatic cancer; **CTC:** Circulating tumor cell; **CEA:** Carcinoembryonic antigen; **CK:** Cytokeratin; **CP:** Chronic pancreatitis; **DAPI:** 4′,6′-diamidino-2- phenylindole; **EpCAM:** Epithelial cellular adhesion molecule; **FISH:** Fluorescence in situ hybridization; **HV:** Healthy volunteer; **ICC:** Immunocytochemistry; **IHC:** Immunohistochemistry; **PB:** Peripheral blood; **PVB:** Portal venous blood; **RT-PCR:** Reverse transcription polymerase chain reaction; **ISET:** Size of Epithelial Tumor; **SE-iFISH:** Immunostaining-fluorescence in situ hybridization; **ALDH:** Aldehyde dehydrogenase; **ZEB1:** Zinc finger E-box binding homebox 1; **PDX-1:** Pancreatic and duodenal homeobox 1; **h-TERT:** Human telomerase reverse transcriptase; **CD:** Cluster of differentiation; **C-MET:** Cellular mesenchymal to epithelial transition factor; **NA:** Not available
